# Electronic structure, thermodynamic stability and high-temperature sensing properties of Er-α-SiAlON ceramics

**DOI:** 10.1038/s41598-020-61105-z

**Published:** 2020-03-18

**Authors:** Yuwaraj K. Kshetri, Takashi Kamiyama, Shuki Torii, Sang Hoon Jeong, Tae-Ho Kim, Heechae Choi, Jun Zhou, Yuan Ping Feng, Soo Wohn Lee

**Affiliations:** 1 Research Center for Eco-Multifunctional Nano Materials, Sun Moon University, Chungnam, 31460 Republic of Korea; 2 Institute of Materials Structure Science, High Energy Accelerator Research Organization J-PARC Center, KEK, 203-1, Tokai, Ibaraki 319-1106 Japan; 3 Department of Environment and Bio-Chemical Engineering, Sun Moon University, Chungnam, 31460 Republic of Korea; 4 Division of Mechanics and ICT Convergence Engineering, Sun Moon University, Chungnam, 31460 Republic of Korea; 5 Institute of Inorganic Chemistry, University of Cologne, 50939 Cologne, Germany; 6 Department of Physics, National University of Singapore, 2 Science Drive 3, 117551 Singapore, Singapore

**Keywords:** Optical materials and structures, Materials science

## Abstract

α-SiAlON ceramics have been in use as engineering ceramics in the most arduous industrial environments such as molten metal handling, cutting tools, gas turbine engines, extrusion molds, thermocouple sheaths, protective cover for high-temperature sensors, etc., owing to their outstanding mechanical, thermal and chemical stability. Taking advantage of the intrinsic properties of α-SiAlONs, we investigate, in this paper, the possibility of using the Er-doped α-SiAlON (Er-α-SiAlON) ceramic as a high-temperature sensing material via its unique near-infrared to visible upconversion property. We first use neutron diffraction and density functional theory calculations to study the electronic structure and thermodynamic stability of Er-α-SiAlON. It is found that the interstitial doping of Er stabilizes the α-SiAlON structure via chemical bonds with O-atoms with N:O ratio of 5:2 in the seven-fold coordination sites of the Er^3+^ ion. Temperature-dependent upconversion emissions are then studied under 980 and 793 nm excitations over a temperature range of 298–1373 K and the fluorescence intensity ratio (FIR) technique has been employed to investigate the temperature sensing behavior. Temperature-dependent Raman behavior is also investigated. We demonstrate that using Er-α-SiAlON as a sensing material, the limit of temperature measurement via the FIR technique can be pushed well beyond 1200 K.

## Introduction

SiAlON ceramics are high-performance refractory ceramics which are manufactured by combining raw materials silicon nitride, alumina, aluminum nitride along with the oxide of rare earth elements. The SiAlON ceramics exist in two basic forms; each form is isostructural with one of the two common forms of Si_3_N_4_. In α-SiAlON, Si in the tetrahedral structure in Si_3_N_4_ is replaced by Al with limited substitution of N by O. Valancy requirements are satisfied by modifying cations occupying the interstitial holes at (1/3, 2/3, z) and (2/3, 1/3, ½ + z) per unit cell of the α-Si_3_N_4_^1^. In this way cations of yttrium (Y), calcium (Ca), lithium (Li), neodymium (Nd), Erbium (Er), etc., for example, can be incorporated into the structure. Consequently, α-SiAlON has the general formula M_x_^v+^Si_12−m−n_ Al_m+n_O_n_N_16−n_ where x = m/v and M is the metal cation^1^. The interstitial dissolution of the M ion stabilizes the α-SiAlON structure and the cations M are coordinated by seven (N, O) atom sites^2,3^.

α-SiAlON ceramics are widely used for high-temperature and high-endurance applications owing to their ability to withstand high structural loads and their excellent thermal and chemical stability^4,5^. Taking advantage of their superior mechanical, thermal and chemical stabilities, these materials have also been investigated recently for the possibility of functional applications such as downshifting phosphor materials for solid-state lighting^6^. In another aspect of investigating the functional properties of these ceramics, we recently reported efficient near-infrared to visible frequency upconversion in lanthanides (Ln^3+^)-doped α-SiAlON ceramics^7–9^. Er-doped α-SiAlON (Er-α-SiAlON) ceramic has moderately low phonon energy and can be a potential upconversion material for high-temperature applications. In one particular application, SiAlON ceramic has been used as protective ceramic coatings for sensing device protection and improving thin-film durability for long term high-temperature application at 1000 °C^10^. But the possibility of using the SiAlON ceramic itself as a high-temperature sensing material via its functional property has not been investigated so far.

It is known that Er^3+^ ion has a thermally coupled pair of energy levels ^2^H_11*/*2_ and ^4^S_3*/*2_, whose green emission intensity varies with temperature. By measuring the fluorescence intensity ratio (FIR) of the two green emission peaks from Er^3+^ ions, it is possible to use it as a probe for measuring environmental temperature where it is inserted^11–14^. The optical temperature sensing based on the FIR technique has attracted considerable attention for high sensitivity and accuracy of the measurement. However, the host materials for the optical temperature sensors are mainly based on glasses and fluorides^15–20^. These glass hosts have relatively poor stability which hampers their application as high-temperature sensors. Moreover, for glasses, the glass transition temperature must be taken into account because it marks the smooth passage of glass to the super-cooled molten state during heating. The glass transition region is the limiting parameter for the temperature range in which the glass temperature sensor can be applied^11^. For example, tellurite glass-based material cannot be used for temperature above 250 °C (523 K) due to its very low transition temperature (350 °C). To overcome these limitations, Er-α-SiAlON ceramic can be a potential candidate for the optical thermometry applications based on the FIR technique. A particular advantage of investigating the Er-α-Sialon as a high-temperature sensor material is that it has outstanding mechanical, thermal and chemical stability even at an elevated temperature above 1000 °C (1273 K)^4^. This offers a very high laser-induced damage threshold which in turn offers high spectral stability of the sensing material.

For the application of Er-α-SiAlON in a high-temperature environment, an understanding of its thermodynamic stability is also essential. In spite of the huge number of experimental investigations of α-SiAlON ceramics, only a few first principles calculations of Ca, Y, and Dy-doped α- and β-SiAlONs have been reported^21–23^. The structures of α-SiAlONs are complex and have not been precisely determined, because the random distribution of O and N leads to fluctuating bond lengths. Cole *et al*.^24^ used the technique of extended X-ray absorption fine structure (EXAFS) spectroscopy in Er-α-SiAlON to investigate the local environment surrounding the Er^3+^ stabilizing cation. The work confirms the seven-fold coordination of the modifying cation within the interstices but indicates a N:O ratio of 5:2 rather than the 6:1 ratio as indicated by the Rietveld refinements of calcium and yttrium containing α-SiAlON by Izumi *et al*.^2,25^. X-ray diffraction technique is not sufficient because the scattering factors of both Si and Al and also O and N are too similar which prevents to obtain precise occupancy information. Similarly, EXAFS suffers the same issue of contrast and is difficult to implement when substitution concentration is low. In contrast to these techniques, neutron diffraction analysis is particularly appropriate to study the preferential occupation in SiAlONs because the lighter elements Si, Al, O and N have considerably different neutron scattering lengths. For example, neutron scattering lengths for O and N are 5.803 fm and 9.360 fm, respectively. Hence neutron diffraction of Er-α-SiAlON in conjunction with first principles calculation can provide greater insight into the local structure and high-temperature stability of this material.

In this work, we present a systematic investigation of thermodynamic stability and electronic structure of Er-α-SiAlON using density functional theory (DFT) calculations. We carried out neutron diffraction experiment to find accurate crystal structure for the electronic structure calculations. We then demonstrate the potential application of Er-α-SiAlON for high-temperature measurement in optical thermometry via the FIR technique. We also investigate the temperature dependence of the Raman spectra of Er-α-SiAlON which has not been reported for SiAlON-based materials.

## Result and Discussion

### Crystal structure from neutron diffraction

**Figure 1:**
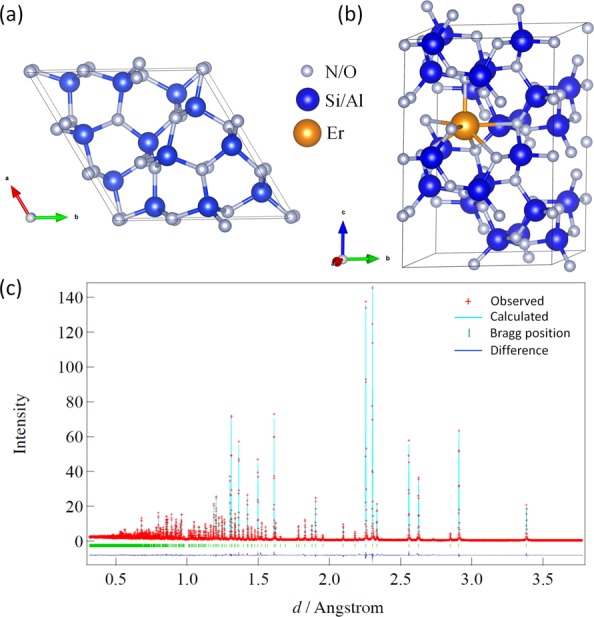
(**a**) Primitive unit cell of α-Si_3_N_4_ viewed along nearly parallel to c vector. (**b**) 1 × 1 × 2 supercell of Er-α-SiAlON. (**c**) Neutron diffraction spectrum of Er-α-SiAlON ceramic.

The Rietveld data fit on the neutron diffraction data of the Er-α-SiAlON ceramic and the difference pattern between simulated and the experimental data are shown in Fig. 1(c). α-SiAlON is the most dominant phase observed. A small amount of impurity phase was identified and was assigned to 12H-SiAlON polytypoids (less than 2%). In hot-press-sintered SiAlONs starting with α-Si_3_N_4_, a trace amount of β-SiAlON usually appears as an impurity phase. However, no β-SiAlON phase was observed in our system. The structural details of the Er-α-SiAlON ceramic as calculated by Rietveld refinement is shown in Supplementary Table S1 online. Taking into account the difference in composition between our sample and by Cao *et al*.^26^, for the neutron diffraction, there is a good agreement in the lattice parameters and atomic positions. The refined crystal structure obtained from the neutron diffraction was used as starting geometry for the DFT calculations.

### Thermodynamic stability and electronic structure

The formation energies of Er, Al and O doped systems were calculated for the chemical reactions below (reactions I to VI). The temperature was set to the sintering temperature of 1850 °C. Solid Er_2_O_3_ was considered as reservoirs of the Er^3+^ dopant in our modeling. The reactions of Al-O codoping, Er-O codoping, and Er-, O- doping are as follow:

(I) Substitution of Al-O co-doping (Al_Si_-O_N_)

$$\frac{{\boldsymbol{x}}}{2}{\boldsymbol{A}}{{\boldsymbol{l}}}_{2}{{\boldsymbol{O}}}_{3}+{\boldsymbol{S}}{{\boldsymbol{i}}}_{3}{{\boldsymbol{N}}}_{4}\to {\boldsymbol{S}}{{\boldsymbol{i}}}_{3-{\boldsymbol{x}}}{\boldsymbol{A}}{{\boldsymbol{l}}}_{{\boldsymbol{x}}}{{\boldsymbol{N}}}_{4-{\boldsymbol{x}}}{{\boldsymbol{O}}}_{{\boldsymbol{x}}}+\frac{{\boldsymbol{x}}}{4}{{\boldsymbol{O}}}_{2({\boldsymbol{g}})}\,\uparrow $$

(II) Interstitial Al^3+^ formation with a triple positive charge (Al_int_^3+^)

$$\frac{{\boldsymbol{x}}}{2}{\boldsymbol{A}}{{\boldsymbol{l}}}_{2}{{\boldsymbol{O}}}_{3}+{\boldsymbol{S}}{{\boldsymbol{i}}}_{3}{{\boldsymbol{N}}}_{4}\to {\boldsymbol{S}}{{\boldsymbol{i}}}_{3}{\boldsymbol{A}}{{\boldsymbol{l}}}_{{\boldsymbol{x}}}{{\boldsymbol{N}}}_{4}+\frac{3{\boldsymbol{x}}}{4}{{\boldsymbol{O}}}_{2({\boldsymbol{g}})}\,\uparrow $$

(III) Substitution of O for N (O_N_)

$$\frac{{\boldsymbol{x}}}{2}{{\boldsymbol{O}}}_{2({\boldsymbol{g}})}+{\boldsymbol{S}}{{\boldsymbol{i}}}_{3}{{\boldsymbol{N}}}_{4}\to {\boldsymbol{S}}{{\boldsymbol{i}}}_{3}{{\boldsymbol{N}}}_{4-{\boldsymbol{x}}}{{\boldsymbol{O}}}_{{\boldsymbol{x}}}+\frac{{\boldsymbol{x}}}{2}{{\boldsymbol{N}}}_{2({\boldsymbol{g}})}\,\uparrow $$

(IV) Interstitial O^2−^ formation with a double negative charge (O_int_^2−^)

$$\frac{{\boldsymbol{x}}}{2}{{\bf{O}}}_{2({\bf{g}})}+{\bf{S}}{{\bf{i}}}_{3}{{\bf{N}}}_{4}\to {\bf{S}}{{\bf{i}}}_{3}{{\bf{N}}}_{4}{{\bf{O}}}_{{\bf{x}}}$$

(V) Substitutional Er-O codoping (Er_Si_-O_N_)

$$\frac{{\boldsymbol{x}}}{2}{\boldsymbol{E}}{{\boldsymbol{r}}}_{2}{{\boldsymbol{O}}}_{3}+{\boldsymbol{S}}{{\boldsymbol{i}}}_{3}{{\boldsymbol{N}}}_{4}\to {\boldsymbol{S}}{{\boldsymbol{i}}}_{3-{\boldsymbol{x}}}{\boldsymbol{E}}{{\boldsymbol{r}}}_{{\boldsymbol{x}}}{{\boldsymbol{N}}}_{4-{\boldsymbol{x}}}{{\boldsymbol{O}}}_{{\boldsymbol{x}}}+\frac{{\boldsymbol{x}}}{4}{{\boldsymbol{O}}}_{2}({\boldsymbol{g}})\,\uparrow $$

(VI) Interstitial Er^3+^ formation with triple positive charge (Er_int_^3+^)

$$\frac{{\boldsymbol{x}}}{2}{\boldsymbol{E}}{{\boldsymbol{r}}}_{2}{{\boldsymbol{O}}}_{3}+{\boldsymbol{S}}{{\boldsymbol{i}}}_{3}{{\boldsymbol{N}}}_{4}\to {\boldsymbol{S}}{{\boldsymbol{i}}}_{3}{\boldsymbol{E}}{{\boldsymbol{r}}}_{{\boldsymbol{x}}}{{\boldsymbol{N}}}_{4}+\frac{3{\boldsymbol{x}}}{4}{{\boldsymbol{O}}}_{2}({\boldsymbol{g}})\,\uparrow $$

**Figure 2:**
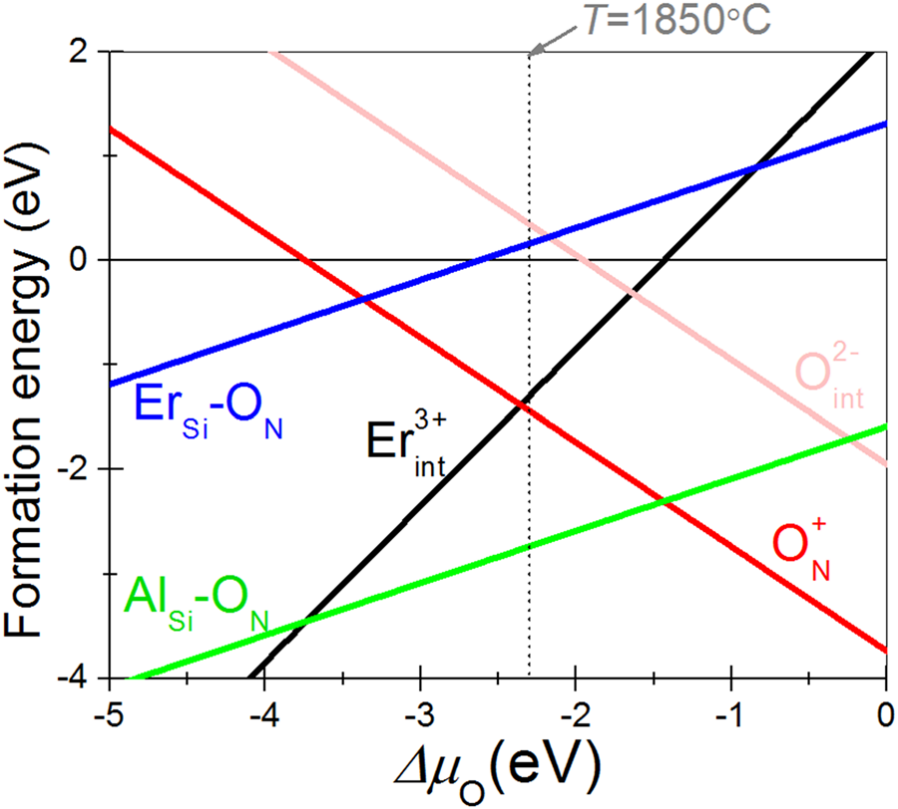
Calculated formation energies of Al-, O- and Er-doping in Si_3_N_4_. The dotted vertical line is consistent with the processing temperature of 1850 °C.

The doping energies of the reactions considered above are plotted as a function of oxygen chemical potential in Fig. 2. The formation energy of interstitial Al-doping (Al_int_^3+^) was much higher than those of all the other doping reactions (5.4 eV/atom for the temperature of 1850 °C) and was not depicted in Fig. 2. It is seen that the substitutional Al-O codoping (Al_Si_-O_N_) is much stable than interstitial Al-doping (Al_int_^3+^), while Er-interstitial doping (Er^3+^_int_) is more stable than substitutional doping of Er along with adjacent O (Er_Si_-O_N_). From the calculated formation energies of Al-, O-, and Er-doping, the most stable configuration of Er-α-SiAlON is obtained by Al-O codoping in substitutional sites and Er^3+^ doping in the interstitial site. It is, therefore, evident that the interstitial doping of Er^3+^ ion, as shown in the model structure in Fig. 1(b), gives thermodynamically the most stable configuration of Er-α-SiAlON. This is in good agreement with the experimental observation that the doped metal cations in α-SiAlON take interstitial positions^1,25^. In the (Al/Si, O/N) doping the Al and O atoms prefer the nearest site (See Supplementary Fig. S1(a) online). In (Er/Si, O/N) substitution, Er and O atoms also prefer the nearest sites as shown in Supplementary Fig. S1(b) online, but this structure is highly unstable because of its higher formation energy (Er_Si_-O_N_) as can be seen from Fig. 2. In addition, neither interstitial O^2−^ doping (O_int_^2−^) nor substitutional O/N doping with one excess positive charge (O_N_^+^) is stable as shown in Fig. 2.

**Figure 3:**
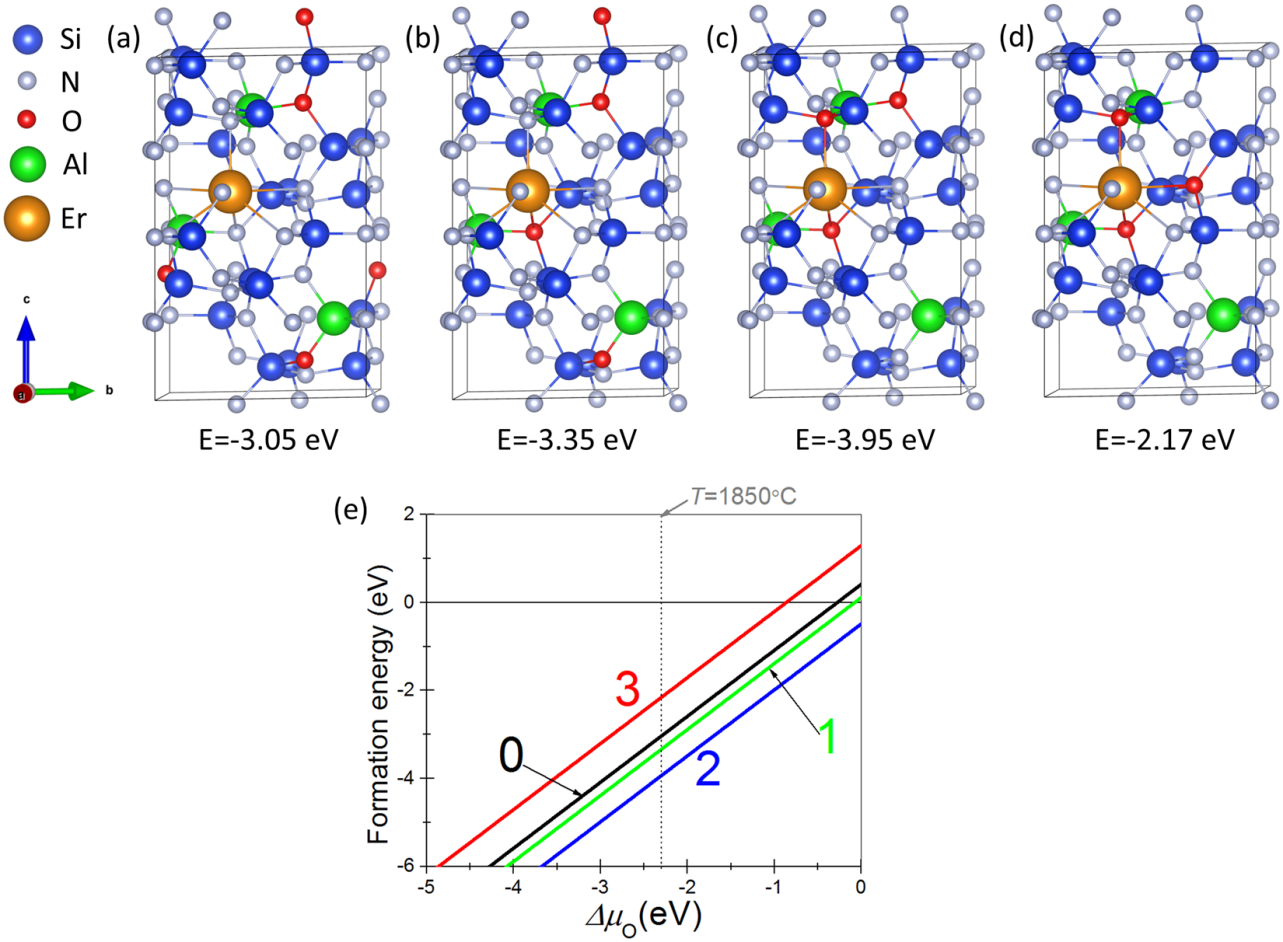
Number of Er–O bonds and formation energies; (**a**) no Er–O bonds, (**b**) one Er–O bond, (**c**) two Er–O bonds, and (**d**) three Er–O bonds, respectively. The model (**c**) is energetically the most stable configuration. (**e**) Formation energies for varying oxygen chemical potential. The curves labeled 0, 1, 2, and 3 correspond to the formation energy of model (**a**), (**b**), (**c**) and (**d**), respectively.

Since Er^3+^ doping into the interstitial site is energetically favorable and Er and O atoms prefer nearest sites, we calculated the formation energies of this configuration for a different number of O atoms surrounding the Er atom as shown in Fig. 3(a–d). The corresponding graph of the formation energies for varying oxygen chemical potential is shown in Fig. 3(e). We see that Er-α-SiAlON is highly stable especially with the atomic structure model shown in Fig. 3(c). That is, the Er-interstitial doping is highly stabilized via chemical bonding with two O-atoms doped in N-sites. This configuration leads to N:O ratio of 5:2 in the seven-fold coordination environment around Er^3+^ ion. This result is in good agreement with the experimental study by Cole *et al*.^24^ of the local environment of the Er^3+^ stabilizing cation in Er-α-SiAlON. According to their work, Er^3+^ stabilizing cation has seven-fold coordination within the interstices but indicates a N:O ratio of 5:2 rather than the 6:1 ratio as indicated by the Rietveld refinements of calcium and yttrium containing α-SiAlON by Izumi *et al*.^2,25^. Hence, our first principles calculation confirms that the stabilizing cation Er^3+^ does occupy the large closed interstices in the (Si, Al)-(N, O) network positioned at (0.333, 0.667, z) and (0.667, 0.333, 0.500 + z) with N:O ratio of 5:2 in the seven-fold coordination sites surrounding the stabilizing cation. Because of the small cation site, the metal cation evidently prefers coordination with smaller O anions than the larger N anions, as compared to that expected from average O/N ratio^27^.

**Figure 4:**
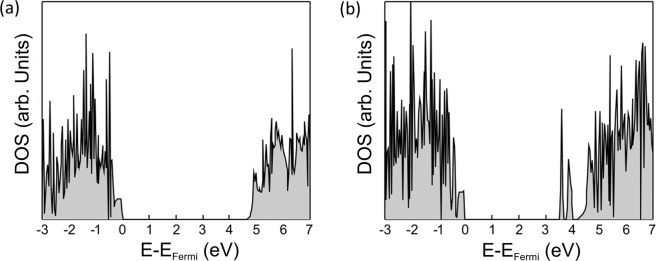
The electron density of states of (**a**) α-SiAlON and (**b**) Er-α-SiAlON, respectively.

The calculated band gap of the pure Si_3_N_4_ is 4.7 eV, which is smaller than the experimental band gap of 5.1 eV of α-Si_3_N_4_^28^. It is well known that GGA underestimates the band gap. Kresse *et al*.^29^ have predicted a much larger band gap of 6.1 eV from self-consistent quasiparticle GW calculations. The density of states (DOS) of the most stable configuration of α-SiAlON and Er-α-SiAlON are shown in Fig. 4(a) and (b), respectively. The two mid-gap states below the conduction band emerge for the most stable configuration of Er-α-SiAlON while they are absent for the α-SiAlON. The mid-gap states in Er-α-SiAlON can, therefore, be attributed to the Er^3+^ ion. The band gap is also reduced to 4.22 eV. This reduction in band gap and the presence of the mid-gap states make Er-α-SiAlON an optically active material.

### Microstructure investigation

**Figure 5:**
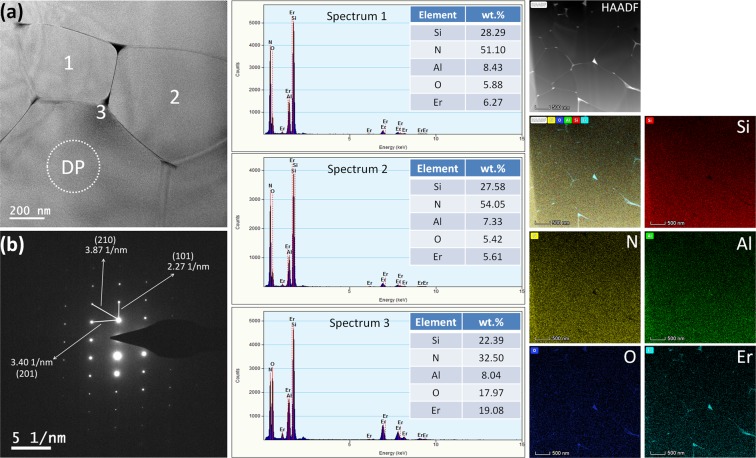
(**a**) TEM micrograph of the Er-α-SiAlON. (**b**) SAED pattern of the region DP in Figure (**a**). The EDS spectra 1, 2 and 3 correspond to the points marked 1, 2 and 3 in Figure (**a**). The elemental mapping diagram of Si, N, Al, O and Er correspond to the region shown in HAADF image.

The TEM micrograph in Fig. 5(a) reveals the grains of α-SiAlON phase in the sintered body. The lattice spacing calculated from the selected area electron diffraction (SAED) pattern, Fig. 5(b), which correspond to the lattice planes (210), (101) and (201) subsequently verifies the α-SiAlON phase. The energy dispersive spectroscopy (EDS) spectra 1 and 2 which correspond to the points inside the α-SiAlON grain indicate that the elemental distributions within different grains are fairly similar. The EDS spectrum 3 corresponding to the triple junction shows a relatively higher Er concentration. It is because in equilibrium situation during sintering, the Er^3+^ ions which cannot be incorporated into the α-SiAlON lattice may segregate in the triple junctions. The high-angle annular dark-field (HAADF) TEM image and corresponding elemental mappings show the homogeneous distribution of elements across the matrix of Er-α-SiAlON. The high-resolution TEM (HRTEM) image of the Er-α-SiAlON has been shown in Supplementary Fig. S2 online. A very thin grain boundary (less than 1 nm) can be seen which indicates effective incorporation of the sintering aids into the α-SiAlON lattice. The lattice spacing of 0.251 and 0.230 nm of the upper and lower grains in the HRTEM image correspond to (210) and (112) crystal planes, respectively, which are also consistent with the α-SiAlON phase.

### Upconversion and temperature sensing property

**Figure 6:**
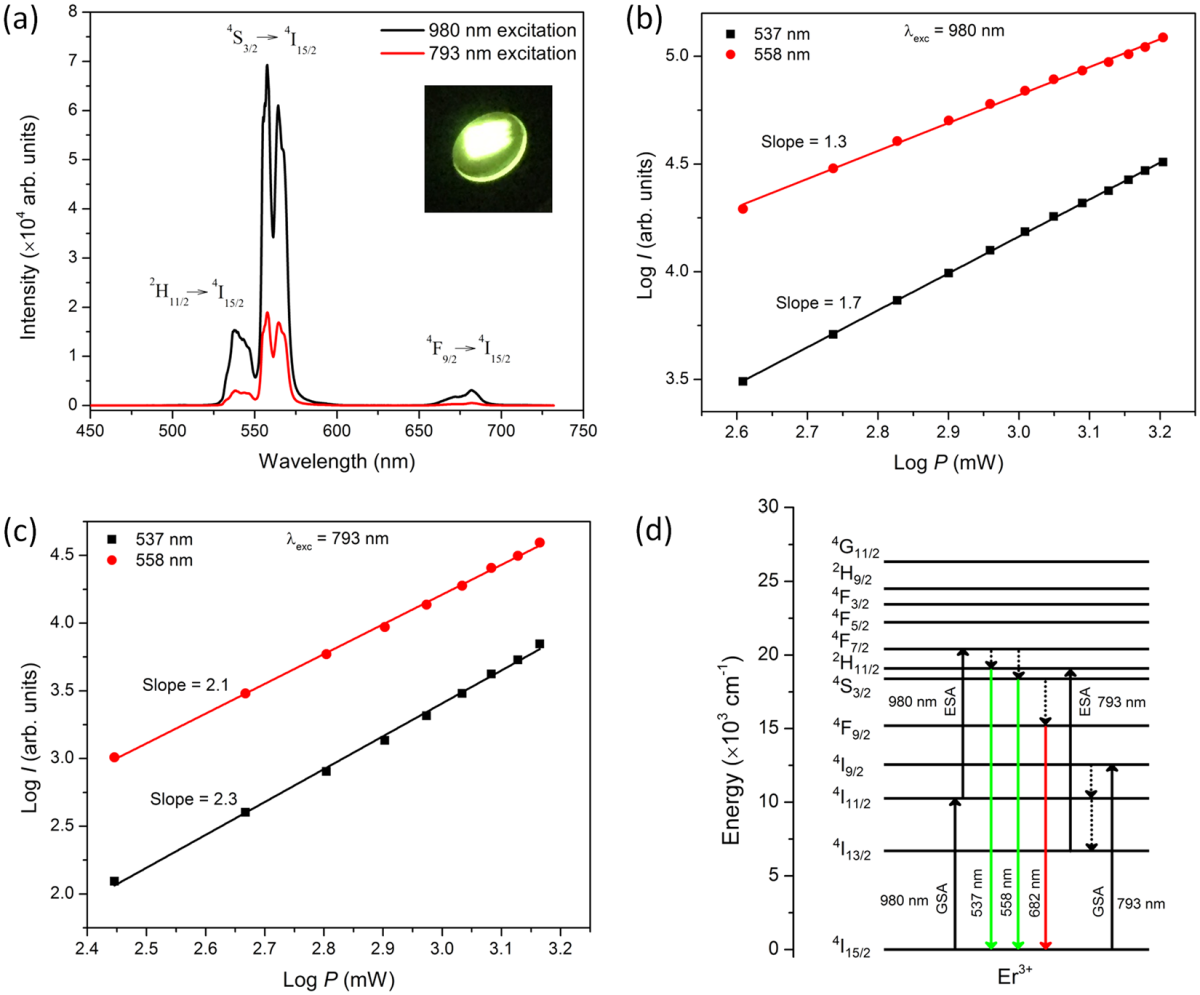
(**a**) Upconversion emission spectra of Er-α-SiAlON under 980 and 793 nm excitations. Inset shows the green emission photograph of Er-α-SiAlON under 980 nm excitation. (**b**) and (**c**) are pump power dependence of the emission intensity in double logarithmic representation. (**d**) Schematic energy level diagram under 980 and 793 nm excitations.

Figure 6(a) shows the upconversion emission spectra of Er-α-SiAlON under 980 and 793 nm excitations measured at room temperature. It can be seen that emission intensity is stronger under 980 nm. The two strong green emission bands centered at 537 and 558 nm are attributed to ^2^H_11/2_ → ^4^I_15/2_ and ^4^S_3/2_ →^4^I_15/2_ transitions while weak red emission band centered at 682 nm is assigned to ^4^F_9/2_ → ^4^I_15/2_ transition of Er^3+^, respectively. For the temperature sensing property, the strong green emission bands are responsible. The pump power dependence of the emission intensity of each green emission band under 980 nm and 793 nm excitations has been shown in Fig. 6(b,c), respectively. Pollnau *et al*.^30^ have shown that the emission intensity *I* is proportional to the *n*^th^ power of the absorbed pump power *P* i. e., ${\boldsymbol{I}}\propto {{\boldsymbol{P}}}^{{\boldsymbol{n}}}\Rightarrow \,\mathrm{Log}({\boldsymbol{I}})\propto {\boldsymbol{n}}\,\mathrm{Log}({\boldsymbol{P}})$ where *n* is the number of pump photons absorbed per upconverted photon and is given by the slope of the emission intensity versus pump power in a double-logarithmic scale. As shown in Fig. 6(b,c) of the plots of the log *I* versus log *P*, the number of photons involved for the two green emission bands at 537 nm and 558 nm under 980 nm excitation are 1.3 and 1.7 while that under 793 nm excitation are 2.1 and 2.3, respectively. Hence, the upconversion mechanism under both laser excitations consists of a two-photon absorption process. Some deviation of *n* from typical values is due to the competition between the linear decay and the upconversion processes for the depletion of the intermediate excited states. Based on this observation, a schematic energy level diagram of Er^3+^ under both 980 and 793 nm excitations has been presented in Fig. 6(d). Two possible mechanisms are responsible for the upconversion emission, viz., ground state absorption (GSA) followed by excited state absorption (ESA) and energy transfer upconversion (ETU) under both the excitation wavelengths. The former involves only one optically active Er^3+^ ion successively promoted to the upper levels by the resonant absorption of two or more laser photons, whereas the latter is a many-body non-radiative mechanism involving two or more nearby interacting Er^3+^ ions. Both mechanisms can co-exist to effectively contribute to the population of the higher excited emitting level^17^. Detail, mechanism of the upconversion process has been investigated under 980 nm and ~800 nm by several researchers^31–33^. Under 980 nm excitation, two successive absorptions of photons bring electrons from the ground state ^4^I_15*/*2_ to the ^4^F_7*/*2_ excited state via the intermediate state ^4^I_11*/*2_. The green-emitting states ^2^H_11*/*2_ and ^4^S_3*/*2_ are then populated by rapid non-radiative relaxations of ^4^F_7*/*2_ level. Upon excitation by 793 nm laser, ^4^I_9*/*2_ level is first excited and quasi-instantaneously the ^4^I_13*/*2_ level is populated through rapid non-radiative relaxations. The ESA process then excites the higher levels ^2^H_11*/*2_ and ^4^S_3*/*2_ giving green emissions as shown in Fig. 6(d).

The energy level ^2^H_11*/*2_ can also be populated from ^4^S_3*/*2_ by thermal excitation due to a very small energy gap between these two levels (≈701 cm^−1^) and therefore these two energy levels are called thermally coupled levels. By measuring the FIR of the two green emission peaks from ^2^H_11/2_ → ^4^I_15/2_ and ^4^S_3/2_ → ^4^I_15/2_ transitions, it is possible to use it as a probe for measuring environmental temperature where it is inserted^34^. The relative population of these thermally coupled levels of Er^3+^ follows a Boltzmann-type population distribution given by Eq. (1)^11,33,35^

1 $${\boldsymbol{FIR}}=\frac{{{\boldsymbol{I}}}_{537}}{{{\boldsymbol{I}}}_{558}}=\frac{{\boldsymbol{N}}{(}^{2}{{\boldsymbol{H}}}_{11/2})}{{\boldsymbol{N}}{(}^{4}{{\boldsymbol{S}}}_{3/2})}=\frac{{{\bf{g}}}_{{\bf{H}}}{{\boldsymbol{\sigma }}}_{{\bf{H}}}{{\boldsymbol{\omega }}}_{{\bf{H}}}}{{{\bf{g}}}_{{\bf{S}}}{{\boldsymbol{\sigma }}}_{{\bf{S}}}{{\boldsymbol{\omega }}}_{{\bf{S}}}}\exp \left(-\frac{\Delta {\boldsymbol{E}}}{{{\boldsymbol{k}}}_{{\boldsymbol{B}}}{\boldsymbol{T}}}\right)={\boldsymbol{C}}\,\exp \left(-\frac{\Delta {\boldsymbol{E}}}{{{\boldsymbol{k}}}_{{\boldsymbol{B}}}{\boldsymbol{T}}}\right)$$

where, N, g, σ and ω are the number of ions, the degeneracy, the emission cross-section, and the angular frequency of the radiative transitions from ^2^H_11/2_ and ^4^S_3/2_ to the ^4^I_15/2_ level, respectively. ΔE is the energy gap between the ^2^H_11/2_ and ^4^S_3/2_ levels, k_B_ is the Boltzmann constant, T is the absolute temperature and ${\boldsymbol{C}}={{\bf{g}}}_{{\bf{H}}}{{\boldsymbol{\sigma }}}_{{\bf{H}}}{{\boldsymbol{\omega }}}_{{\bf{H}}}/{{\bf{g}}}_{{\bf{S}}}{{\boldsymbol{\sigma }}}_{{\bf{S}}}{{\boldsymbol{\omega }}}_{{\bf{S}}}$ is a constant.

Above equation can also be expressed as

2 $$\mathrm{ln}({\boldsymbol{FIR}})=-\,\left(\frac{\Delta {\boldsymbol{E}}}{{{\boldsymbol{k}}}_{{\boldsymbol{B}}}}\right)\frac{1}{{\boldsymbol{T}}}+\,\mathrm{ln}\,{\boldsymbol{C}}$$

**Figure 7:**
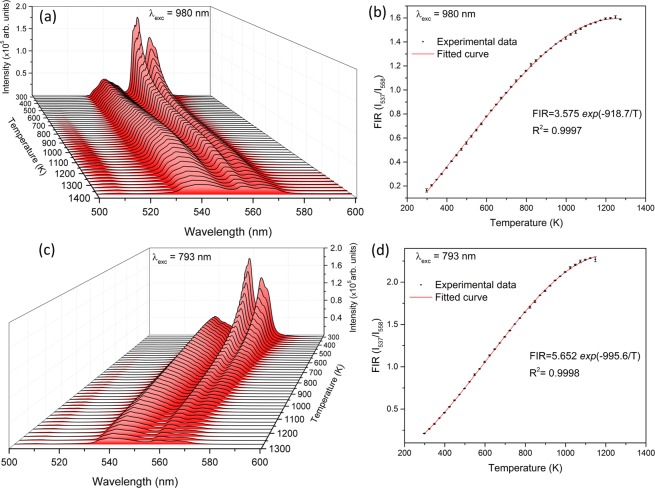
(**a**) Temperature-dependent emission spectra of Er-α-SiAlON under 980 nm excitation. (**b**) Plot of FIR of the two green emissions as a function of temperature in (**a**). (**c**) Temperature-dependent emission spectra of Er-α-SiAlON under 793 nm excitation. (**d**) Plot of FIR of the two green emissions as a function of temperature in (**c**).

**Figure 8:**
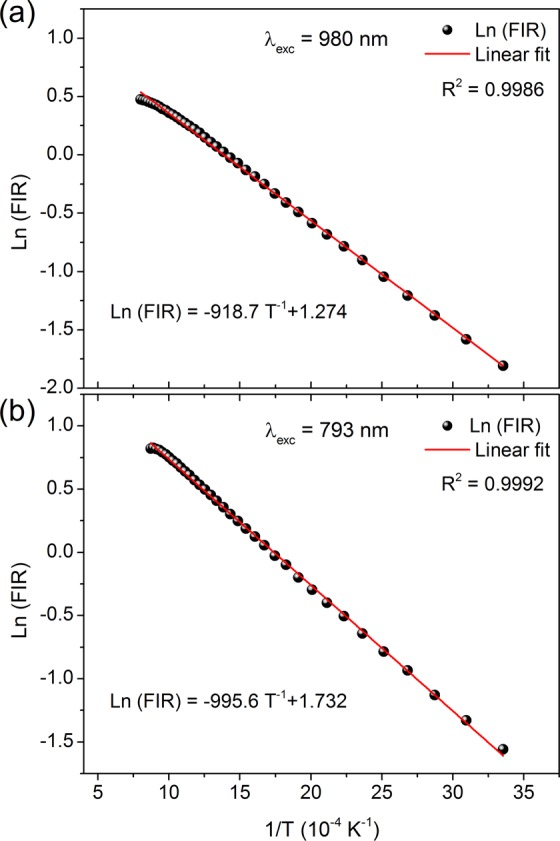
Logarithmic dependence of FIR on inverse temperature (**a**) under 980 nm excitation and (**b**) under 793 nm excitation.

Figure 7(a) shows the temperature-dependent emission spectra under 980 nm excitation in the range of 298–1373K, the highest temperature range investigated for the optical thermometry via the FIR technique. Figure 7(b) shows the FIR of the green upconversion emissions at 537 nm and 558 nm as a function of temperature under 980 nm excitation. The FIR increases from 0.164 at 298 K to 1.61 at a temperature as high as 1248 K. The FIR could be measured up to temperature 1373 K but it decreased above 1248 K because at very high temperature multiphonon decay decreases the luminescence signal. Figure 8(a) shows the linear fit of the experimental data in Eq. (2) from which we obtain the values C = 3.575 and ΔE = 638 cm^−1^. Figure 7(c) shows the temperature-dependent emission spectra under 793 nm excitation and the corresponding FIR of the green upconversion emissions as a function of temperature is shown in Fig. 7(d). The FIR increases from 0.211 at 298 K to 2.27 at a temperature as high as 1123 K. In the case of 793 nm excitation, the FIR decreased above 1123 K because at very high temperature multiphonon decay decreases the luminescence signal. Figure 8(b) shows the linear fit of the experimental data in Eq. (2) from which we obtain the values C = 5.652 and ΔE = 692 cm^−1^. It is emphasized that the values of ΔE obtained by the linear fit under 980 and 793 nm excitations are in very good agreement (less than 9%) with that obtained from the emission spectrum indicating that the sensor sensitivity does not vary significantly. The FIR shows a high degree of linearity with respect to temperature under 793 nm excitation as compared to 980 nm excitation. Repeated experiments were carried out for the temperature-dependent emission spectra and FIR during heating and cooling cycles under 980 and 793 nm laser excitations. The results have been presented in Supplementary Fig. S3 online and Supplementary Fig. S4 online for 980 and 793 nm excitations, respectively. Under each laser excitation, it was found that the emission spectra follow identical emission patterns with only a slight change in the peak intensity during heating and cooling cycles. The FIR also shows highly consistent temperature dependency in both heating and cooling cycles. The result is the indication of high spectral stability and reproducibility of the temperature sensing behavior of Er-SiAlON ceramics.

The absolute thermal sensitivity *S* of the optical temperature sensing material is defined as the rate at which the FIR changes with the temperature of the host matrix^34,35^ i.e.,

3 $${\boldsymbol{S}}=\frac{{\boldsymbol{d}}({\boldsymbol{FIR}})}{{\boldsymbol{dT}}}={\boldsymbol{FIR}}\left(\frac{\Delta {\boldsymbol{E}}}{{{\boldsymbol{k}}}_{{\boldsymbol{B}}}{{\boldsymbol{T}}}^{2}}\right)$$

For the comparison between the sensitivities obtained for the FIR of different lanthanide ion and the host matrix combination, a relative sensitivity *S*_*R*_ is defined as^36^.

4 $${{\boldsymbol{S}}}_{{\boldsymbol{R}}}=\frac{1}{{\boldsymbol{FIR}}}\frac{{\boldsymbol{d}}({\boldsymbol{FIR}})}{{\boldsymbol{dT}}}=\frac{\Delta {\boldsymbol{E}}}{{{\boldsymbol{k}}}_{{\boldsymbol{B}}}{{\boldsymbol{T}}}^{2}}$$

**Figure 9:**
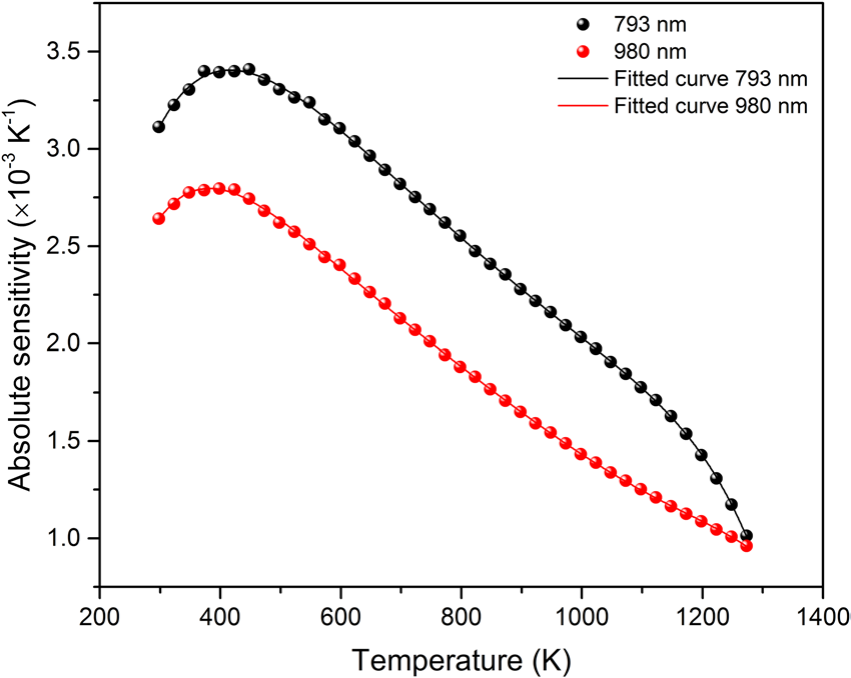
Absolute sensitivity versus temperature plots under 793 and 980 nm excitations.

Usually, absolute maximum sensitivity is employed for comparison among different host materials; however, *S*_*R*_ has the advantage of being independent of the nature of the thermometric technique. The plots of absolute sensitivity versus temperature under 980 and 793 nm excitations have been shown in Fig. 9. Above 1200 K, the sensitivity decreases rapidly under 793 nm excitation as compared to that under 980 nm excitation. The values of the maximum sensitivities, the temperature for the maximum sensitivity and relative sensitivity of Er-α-SiAlON along with the values for different host materials found in literature have been presented in Table 1. In the temperature range of 298–1373 K, we see that the Er-α-SiAlON ceramic shows favorable results. Er-α-SiAlON ceramic has a maximum sensitivity of 3.4 × 10^−3^ K^−1^ at 448 K and a relative sensitivity of 0.59% K^−1^ under 793 nm excitation. While under 980 nm excitation, maximum sensitivity is 2.8 × 10^−3^ K^−1^ at 400 K and *S*_*R*_ is 0.67% K^−1^. It is obvious that the sensitivity is not an intrinsic parameter of the sensing materials but depends on the excitation wavelength. We see that *S*_*R*_ is the highest for Er-α-SiAlON ceramic as compared to other hosts under 980 nm excitation as well as 793 nm excitation. Manzani *et al*. reported a high absolute sensitivity of 8.9 × 10^−3^ K^−1^ in Er/Yb-tellurite glass^11^. However, its maximum operating temperature is limited to just 473 K. Most of the works on optical thermometry via upconversion are focused on the chalcogenide^37^ and fluoride^17^ glasses owing to their low phonon energy. But a significant disadvantage in these materials is that their low transition temperature (T_g_) severely limits the sensor operating temperature although they have higher sensitivity. It can be seen that the sensitivity of Er-α-SiAlON ceramic is better than that of Er-Silicate glass (2.3 × 10^−3^ K^−1^ at 296 K)^38^, Er-oxyfluoride glass (2.7 × 10^−3^ K^−1^ at 543 K)^39^, and it is much higher than that for Er-Na_5_Gd_9_F_32_ glass ceramics (1.7 × 10^−3^ K^−1^ at 499 K)^40^. Even at 1273 K, it can be seen from Fig. 9 that the sensitivity of Er-α-SiAlON ceramic is about 1.0 × 10^−3^ K^−1^ under 793 nm and 980 nm excitations. This sensitivity of Er-α-SiAlON ceramic at 1273 K is still higher than the maximum sensitivity of Er-fluorozirconate glass (0.6 × 10^−3^ K^−1^) at 300 K^13^. Dong *et al*.^41^ reported optical thermometry in Er/Yb-Al_2_O_3_ ceramics capable of operating at 973 K. But our results show that using Er-α-SiAlON ceramics as the sensing material, the limit of the temperature measurement via optical thermometry can be pushed beyond 1200 K, the highest temperature measurement via the FIR technique.

**Table 1:** Comparison of the temperature sensing properties of Er-α-SiAlON ceramics with other materials.

Sensing materials	Maximum absolute sensitivity (S_max_) × 10^−3^ K^−1^	Relative sensitivity (S_R_) % K^−1^	Temperature at maximum sensitivity K	Temperature range (K)	Excitation wavelength (nm)	Reference
Er-α-SiAlON ceramic	3.4	0.59	448	298–1273	793	This work
Er-α-SiAlON ceramic	2.8	0.67	400	298–1373	980	This work
Er-Silicate glass	2.3	—	296	296–673	978	Li *et al*.^38^
Er-Fluorotellurite glass	5.4	0.35	547	300–550	800	Sergio *et al*.^17^
Er-Fluorozirconate glass	0.6	0.58	300	150–850	805	Cai *et al*.^13^
Er/Yb-Al_2_O_3_	5.1	0.40	491	295–973	978	Dong *et al*.^41^
Er/Yb-tellurite glass	8.9	0.53	473	278–473	980	Manzani *et al*.^11^
Er-PLZT ceramic	4.0	—	556	300–883	980	Camargo *et al*.^14^
Er-PbO-Ga_2_O_3_-SiO_2_ glass	2.6	0.24	590	296–650	980	Pisarski *et al*.^20^
Er/Yb-TeO_2_-WO_3_ glass	2.6	0.20	417	300–690	980	Pandey *et al*.^19^
Er-oxyfluoride glass	2.7	0.41	543	250–450	980	Feng *et al*.^39^
Er-fluoroindate galss	2.8	0.55	425	123–425	980	Gonzalez *et al*.^15^
Er-Chalcogenide glass	5.2	—	493	293–493	1060	Santos *et al*.^37^
Er-Na_5_Gd_9_F_32_ glass ceramics	1.7	—	499	300–500	980	Li *et al*.^40^

### Temperature-dependent Raman spectra

**Figure 10:**
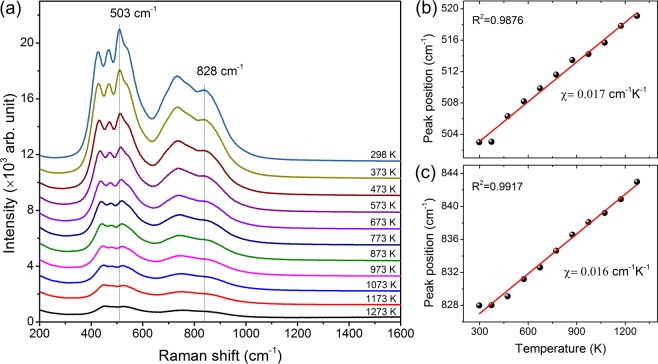
(**a**) Temperature-dependent Raman spectra of Er-α-SiAlON. (**b**) and (**c**) are the Raman shifts of the peaks at 503 and 828 cm^−1^, respectively, as a function of temperature.

For the application of Er-α-SiAlON ceramic as a high-temperature sensing material, understanding of its temperature-dependent phonon behavior is very essential. The comprehensive analysis of the temperature-dependent Raman spectra of Er-α-SiAlON measured for the first time over the temperature range of 298–1273 K has been presented in Fig. 10(a–c). It is found that the most intense peak at 503 cm^−1^ and the highest-phonon energy peak at 828 cm^−1^ are found to be blue-shifted (hardening behavior) in the specified temperature range. Moreover, the overall intensity of the Raman peaks also decreases with increasing temperature. The band positions versus temperatures were fitted using the following equation:

5 $${\boldsymbol{\omega }}({\boldsymbol{T}})={{\boldsymbol{\omega }}}_{{\boldsymbol{o}}}+{\boldsymbol{\chi }}{\boldsymbol{T}}$$

where ${{\boldsymbol{\omega }}}_{{\boldsymbol{o}}}$ is the frequency of vibration at absolute zero temperature and ${\boldsymbol{\chi }}$ is the first-order temperature coefficient which is given by the slope of the fitted line. In Fig. 10(b,c), the values of ${\boldsymbol{\chi }}$ for the peaks at 503 and 828 cm^−1^ are 0.017 and 0.016 cm^−1^K^−1^, respectively. The extrapolated ${{\boldsymbol{\omega }}}_{{\boldsymbol{o}}}$ are 498 and 822 cm^−1^ for the most intense peak and the highest-phonon energy peak respectively. Our result provides new reference data for the first-order temperature coefficients of Er-α-SiAlON ceramics. The shift of Raman frequency with respect to temperature has been reported in other bulk and nanomaterials^42–45^ and it is the manifestation of the anharmonic term in the lattice potential energy. For each vibrational mode, the Raman shift dependence on temperature can be attributed to two reasons; the optical-phonon anharmonic coupling and the thermal expansion. The thermal expansion itself is a result of anharmonicity but it has a different physical mechanism related to the change of force constant with volume. The Raman shift direction varies for different materials, even for a given material the change in phonon frequency may differ for different vibrational modes. For example, the phonon frequencies of two of the E_g_ modes blue-shift while that of B_1g_ mode red-shift with the increase of temperature in anatase TiO_2_^46^. Our observation of the blue- shift of the phonon frequencies in Er-α-SiAlON ceramic indicates that anharmonic couplings of the optical phonons become much stronger with increasing temperature than the contribution arising from thermal expansion. This hardening behavior might be responsible for the reduced luminescence signal observed at high temperatures (above 1248 K) in Er-α-SiAlON ceramics. A detail explanation of the hardening behavior of vibrational modes with increasing temperature in Er-α-SiAlON ceramics can be a strong motivation for further research.

## Conclusion

Combining neutron diffraction, density functional theory calculations and thermodynamic modeling, we have shown that Er doping in the interstitial site of silicon nitride gives thermodynamically the most stable structure of Er-α-SiAlON. Moreover, the interstitial doping of Er is highly stabilized via chemical bonds with O-atoms with N:O ratio of 5:2 in the seven-fold coordination sites surrounding the stabilizing cation Er^3+^. Temperature dependence of the fluorescence intensity ratio of green upconversion emission from thermally coupled energy levels ^2^H_11/2_ and ^4^S_3/2_ of Er^3+^ ion in Er-α-SiAlON under near-infrared excitations has been investigated for the application of this material as a high-temperature sensor. Er-α-SiAlON ceramic has a maximum sensitivity of 3.4 × 10^−3^ K^−1^ and the relative sensitivity of 0.59% K^−1^ under 793 nm excitation while under 980 nm excitation, maximum sensitivity is 2.8 × 10^−3^ K^−1^ and relative sensitivity is 0.67% K^−1^. As the Er-α-SiAlON ceramic offers superior thermal and chemical stability over chalcogenide and fluoride glass-based optical temperature sensors, a maximum operating temperature of 1273 K, the highest temperature measurement via FIR technique in optical thermometry, can be achieved. We observed a blue- shift of the phonon frequencies in Er-α-SiAlON ceramic over the temperature range of 298–1273 K indicating stronger anharmonic couplings of the optical phonons at high temperature.

## Methods

### Model structures

For the simulation of thermodynamic stability and electronic structure of Er-α-SiAlON, a 1 × 1 × 2 supercell of α-Si_3_N_4_ (space group P31c) containing 24 Si atoms and 32 N atoms was used. Both the primitive cell and the 1 × 1 × 2 supercell are displayed in Fig. 1(a,b) respectively. All the drawings of the crystal structure were produced using VESTA software^47^. We took the experimentally determined geometry of Er-α-SiAlON obtained from neutron diffraction as a start point to construct the other model system. In the model structures, the Al/Si substitutions are distributed over the Si_3_N_4_ framework. The lanthanide ion (Er^3+^) in α-SiAlON occupies interstitial sites of the silicon nitride structure. But, in order to investigate the thermodynamic stability, we considered Er/Si substitutions in addition to the interstitial substitution of Er^3+^. Moreover, we considered N/O substitution as well as the interstitial substitution of O in the silicon nitride structure. One Er^3+^ cation per primitive cell of α-Si_3_N_4_ leads to the composition ErSi_9.0_Al_3.0_N_16.0_ with a much higher Er-concentration than in real systems^1^. For a realistic composition, we considered one (Er^3+^ + 3Al/3Si) substitutions per 1 × 1 × 2 supercell.

### Calculation methods

Density functional theory (DFT)^48,49^ calculations were performed using the generalized gradient approximation (GGA), with Perdew-Burke-Ernzerhof (PBE) parameterization^50,51^. We used the Vienna *Ab initio* Simulation Package (VASP) program^52–55^. Atomic nuclei and core electrons were described by a projector-augmented wave (PAW) method. A Hubbard *U* approximation term was used to describe the strongly correlated *d* and *f-*orbitals of the Er^3+^ ions. Khon-Sham orbitals were expanded with a cutoff energy of 400 eV, and a 3 × 3 × 2 and 6 × 6 × 4 equally spaced *k*-point grids were employed for the Brillouin zone sampling in structural relaxations and electronic structure calculations, respectively^56^. Full relaxation of all the atomic positions was performed via a conjugate-gradient algorithm. The stopping criterion of the convergence procedure is a residual force acting on atoms smaller than 0.01 eV/A^3^.

Formation energies, $\Delta {E}_{{\rm{f}}}$ of Al-, O- and Er-doping in Si_3_N_4_ base material are expressed as

6 $$\Delta {E}_{{\rm{f}}}=E({{\rm{D}}}^{q})-{E}_{{{\rm{Si}}}_{3}{{\rm{N}}}_{4}}\pm {\mu }_{{\rm{i}}}$$

where *E*(D^q^) and ${E}_{{{\rm{Si}}}_{3}{{\rm{N}}}_{4}}$ are the calculated total energies of doped and pure Si_3_N_4_, and *μ*_i_ is the chemical potential of species *i*. In the reactions of Al-, O- and Er-doping in Si_3_N_4_, the reservoirs are regarded as Al_2_O_3_, O_2_, and Er_2_O_3_ because those phases are stable in our experimental conditions. The entropy terms of solid phases, Si_3_N_4_, Al_2_O_3_, and Er_2_O_3_ were not considered in the formation free energy calculations since the contributions of entropy to free energy are less than 0.1 eV and are mostly canceled out. For varying temperature and pressure, we express the formation energy (Eq. (6)) as a function of chemical potentials of oxygen and nitrogen,

7 $${\mu }_{{\rm{O}}}(T,{P}_{{{\rm{O}}}_{2}})=\frac{1}{2}\{{\tilde{\mu }}_{{{\rm{O}}}_{2}}(T,{P}^{^\circ })+{k}_{{\rm{B}}}T\,\mathrm{ln}(\frac{{P}_{{{\rm{O}}}_{2}}}{{P}^{^\circ }})\}$$

8 $${\mu }_{{\rm{N}}}(T,{P}_{{{\rm{O}}}_{2}})=\frac{1}{2}\{{\tilde{\mu }}_{{{\rm{N}}}_{2}}(T,{P}^{^\circ })+{k}_{{\rm{B}}}T\,\mathrm{ln}(\frac{{P}_{{{\rm{N}}}_{2}}}{{P}^{^\circ }})\}$$

where ${\tilde{\mu }}_{{{\rm{O}}}_{2}}(T,{P}^{^\circ })$ and $\{{\tilde{\mu }}_{{{\rm{N}}}_{2}}(T,{P}^{^\circ })$ are the oxygen and nitrogen chemical potential at temperature *T* and standard pressure (${P}^{^\circ }$).

### Materials and synthesis

Er-α-SiAlON ceramic was prepared from high purity α-Si_3_N_4_ (SN-E10; UBE Co., Tokyo, Japan), AlN (Grade F; Tokuyama Co., Tokyo, Japan), Al_2_O_3_ (High purity chemicals Co LTD., Osaka, Japan), Er_2_O_3_ (High purity chemicals Co LTD., Tokyo, Japan). A single composition of Er-α-SiAlON was prepared according to the formula M_x_^v+^Si_12−m−n_ Al_m+n_O_n_N_16−n_ with m = 1.5, n = 1.0. The powder preparation and the sintering technique was the same as described in our previous work^7^. The sample was sintered at 1850 °C (2123 K). The sintered samples were ground, cut and mirror-polished for various measurements.

### Characterization

The powder neutron diffraction experiment was carried out at Japan Proton Accelerator Research Complex (J-PARC), Japan, using Super High Resolution Powder Diffractometer (SuperHRPD)^57^. The measurement was done at room temperature for 24 hours. The diffraction data were used for the Rietveld refinement using Z-Rietveld software^58,59^. Microstructure and elemental analysis were carried out using transmission electron microscopy (TEM, Titan Themis Z, FEI, USA) operated at 300 kV. Photoluminescence spectra under 980 nm and 793 nm continuous wave laser (CNI lasers) excitation were recorded by Shamrock spectrograph (Shamrock 303i; Andor). For the measurement of temperature-dependent photoluminescence, Linkam thermal stage (TS1200, Linkam Scientific, UK) was home-customized and integrated with the Shamrock spectrograph. The photoluminescence spectra from 25 to 1100 °C (298 to1373K) were measured in a step of 25 °C under ambient laboratory environment. The incident laser power was fixed at 500 mW. The heating rate was 5 °C/min and the temperature was kept constant for 10 minutes at each step to ensure correct temperature across the sample. The temperature stability was ±1 °C. Temperature-dependent Raman spectra were measured using Raman spectrometer (LabRam HR; Horiba Scientific) coupled with the same Linkam thermal stage as that was used for the temperature-dependent photoluminescence measurement. The sample was excited with 532 nm laser and the spectra were measured from 25 to 1000 °C (298 to1273K) in an ambient laboratory environment.

## Supplementary information

Supplementary information.
