# Supplementary material for: Electronic structure, thermodynamic stability and high-temperature sensing properties of Er-α-SiAlON ceramics
Source: Sci Rep. 2020 Mar 18;10:4952. doi: 10.1038/s41598-020-61105-z (PMC7080770; doi:10.1038/s41598-020-61105-z)

Supplementary Information

Electronic structure, thermodynamic stability and high-temperature sensing properties of Er-α-SiAlON ceramics

Yuwaraj K. Kshetri[1,*], Takashi Kamiyama[2], Shuki Torii[2], Sang Hoon Jeong[3], Tae-Ho Kim[4],

Heechae Choi[5], Jun Zhou[6], Yuan Ping Feng[6], Soo Wohn Lee[1,3,*]

[1]Research Center for Eco-Multifunctional Nano Materials, Sun Moon University, Chungnam 31460, Republic of Korea

[2] Institute of Materials Structure Science, High Energy Accelerator Research Organization (KEK), 203-1, Tokai, Ibaraki 319-1106, Japan
J-PARC Center, KEK, 203-1, Tokai, Ibaraki 319-1106, Japan

[3] Department of Environment and Bio-Chemical Engineering, Sun Moon University, Chungnam 31460, Republic of Korea

[4] Division of Mechanics and ICT Convergence Engineering, Sun Moon University, Chungnam 31460, Republic of Korea

[5] Institute of Inorganic Chemistry, University of Cologne, 50939, Cologne, Germany

[6] Department of Physics, National University of Singapore, 2 Science Drive 3, 117551 Singapore, Singapore

*Corresponding authors: yuwarajkhatri@gmail.com, swlee@sunmoon.ac.kr

Table S1. Results of Rietveld refinement for Er-α-SiAlON ceramic with m=1.5, n= 1.0.

Phase= α-SiAlON

Space group= P31c

Lattice parameters: a= b=7.8145420(6), c=5.6988144(10), α=β= 90°, γ=120°

R_{wp} = 5.87%, R_p = 3.04%, R_e = 0.721%, χ^2 = 6.63

Atom No.	Symbol	Occupancy	x	y	z	B (Å)
1	Er	0.162	1/3	2/3	0.2480 (3)	1.326(19)
2	Si	0.819	0.50942(3)	0.08173(3)	0.21198(3)	0.593(3)
3	Al	0.181	= *x* (Si2)	= *y* (Si2)	= *z* (Si2)	= *B* (Si2)
4	Si	0.919	0.16870(2)	0.25177(2)	0.00188(4)	0.444(2)
5	Al	0.081	= *x* (Si4)	= *y* (Si4)	= *z* (Si4)	= *B* (Si4)
6	N	0.815	0	0	-0.00007(4)	0.4426(18)
7	O	0.185	= *x* (N6)	= *y* (N6)	= *z* (N6)	= *B* (N6)
8	N	0.720	1/3	2/3	0.64958(3)	0.755(3)
9	O	0.280	= *x* (N8)	= *y* (N8)	= *z* (N8)	= *B* (N8)
10	N	0.835	0.343084(11)	-0.047933(10)	-0.016172(19)	0.5763(11)
11	O	0.165	= *x* (N10)	= *y* (N10)	= *z* (N10)	= *B* (N10)
12	N	0.825	0.318718(12)	0.317243(13)	0.24737(2)	0.5799(11)
13	O	0.175	= *x* (N12)	= *y* (N12)	= *z* (N12)	= *B* (N12)

Figure S1. Site preferences of the dopants. (a) (Al/Si, O/N) substitutional doping. Doping with Al and O atoms as nearest sites is energetically more stable. (b) Although (Er/Si, O/N) substitutional doping is not stable, Er and O atoms prefer nearest sites.

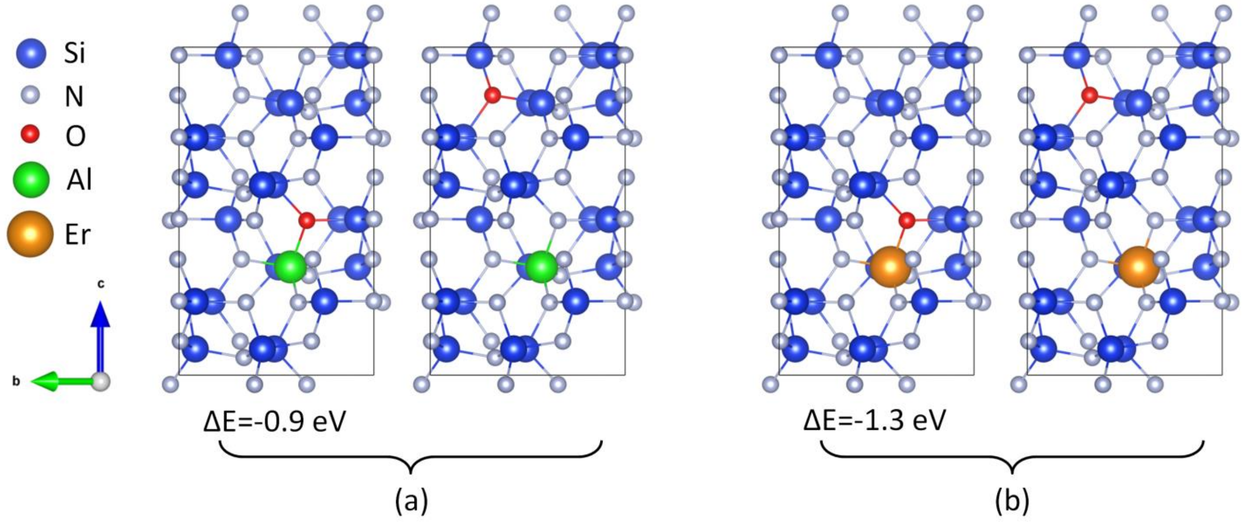

Figure S2. (a) HRTEM image of the Er-α-SiAlON. (b) and (c) are the magnified view of the top and bottom grains in (a).

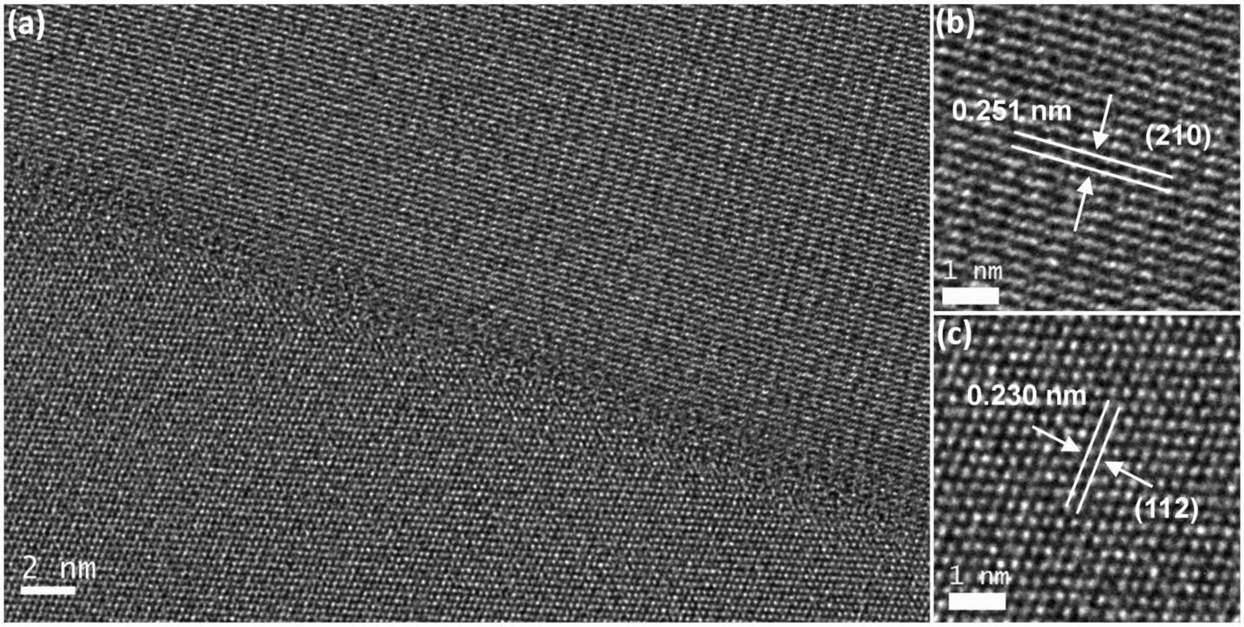

Figure S3. Temperature dependent emission spectra of Er-α-SiAlON under 980 nm excitation. (a) During heating cycle. (b) During Cooling cycle. (c) Plot of FIR of the two green emissions as a function of temperature corresponding to heating (a) and cooling (b) cycles, respectively.

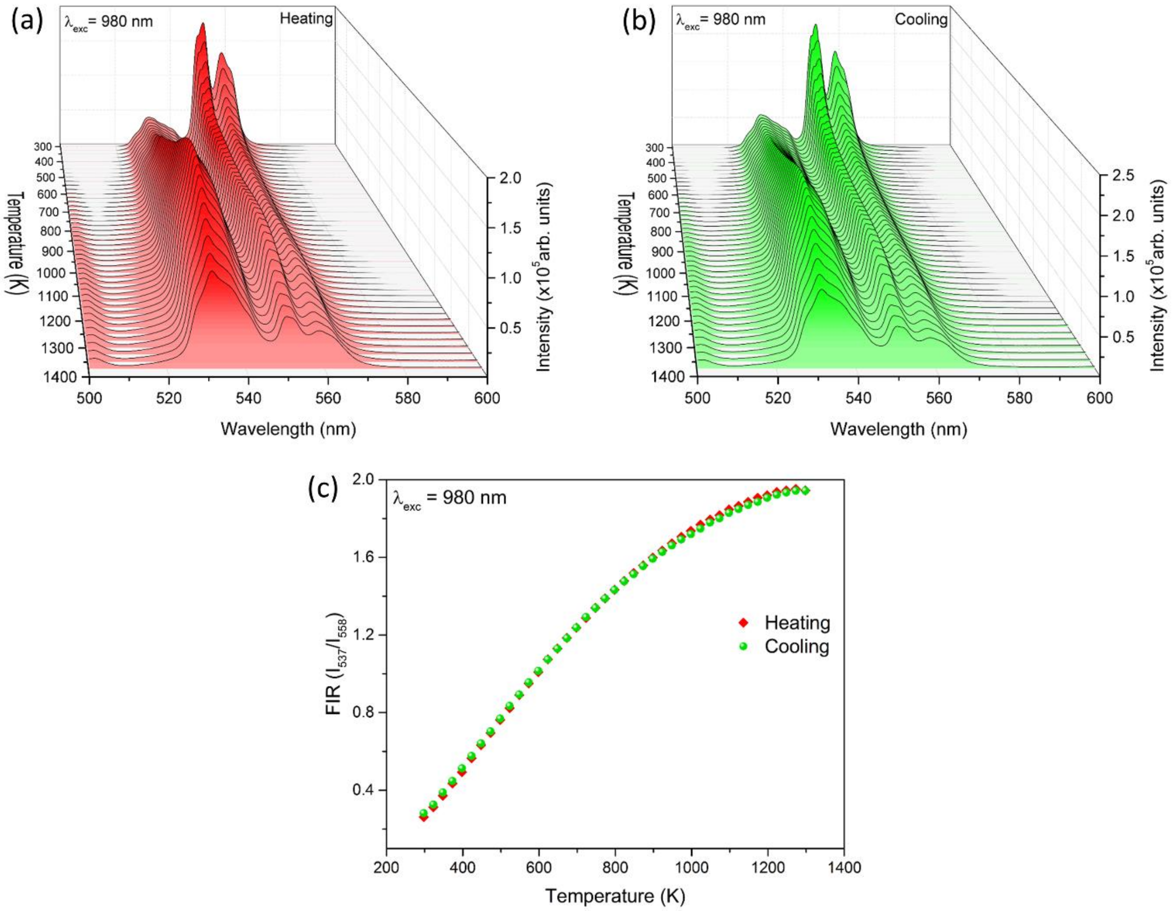

Figure S4. Temperature dependent emission spectra of Er-α-SiAlON under 793 nm excitation. (a) During heating cycle. (b) During Cooling cycle. (c) Plot of FIR of the two green emissions as a function of temperature corresponding to heating (a) and cooling (b) cycles respectively.

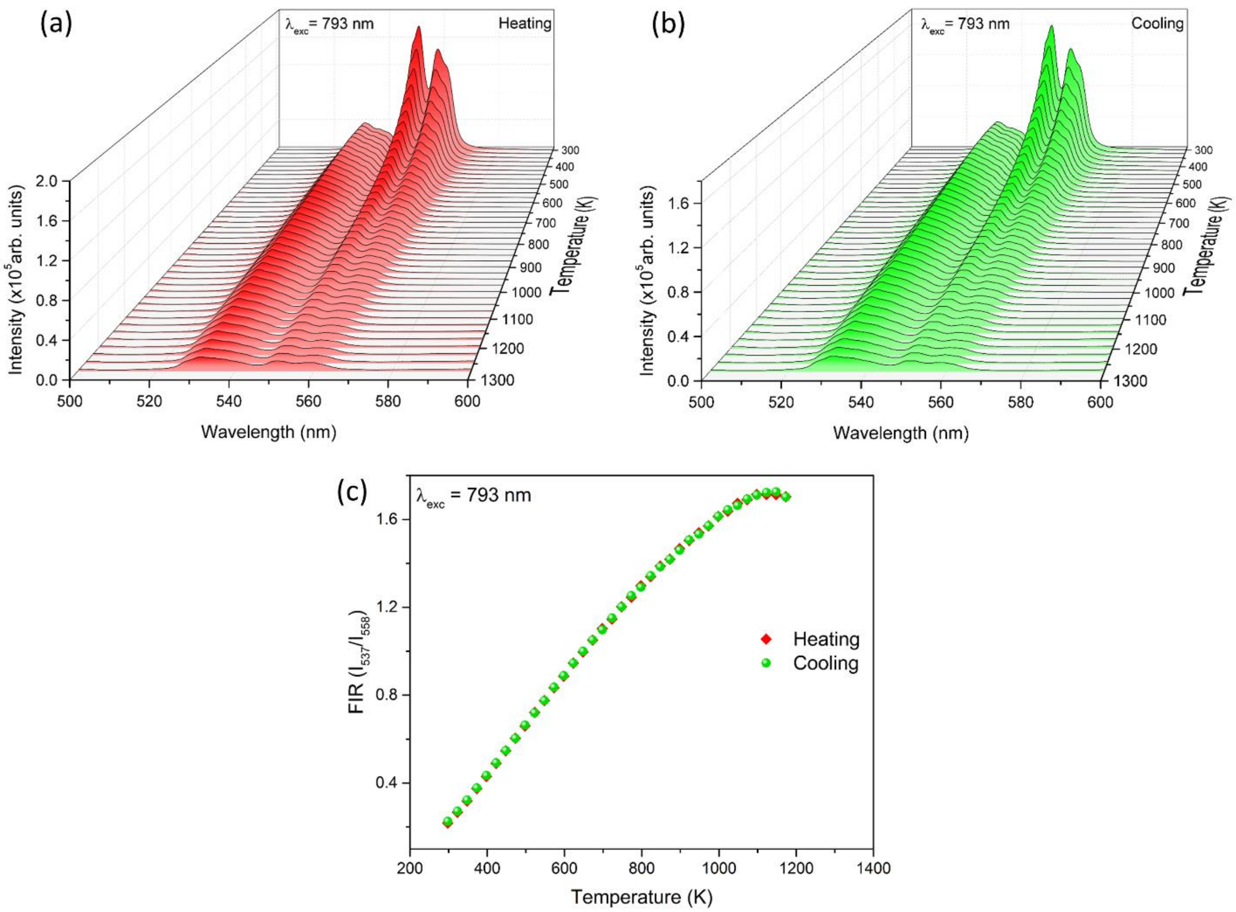

Supplement: Supplementary file 1 — Supplementary information. [file 41598_2020_61105_MOESM1_ESM.pdf]
